# Finite element analysis of femoral neck strains during stair ascent and descent

**DOI:** 10.1038/s41598-021-87936-y

**Published:** 2021-04-28

**Authors:** Chen Deng, Jason C. Gillette, Timothy R. Derrick

**Affiliations:** 1 Division of Sport Biomechanics, School of Sport Science, Beijing Sport University, Beijing, 100084 People’s Republic of China; 2 Department of Kinesiology, Iowa State University, Ames, IA 50010 USA

**Keywords:** Bone, Risk factors, Lifestyle modification

## Abstract

For older population, a better understanding of the hip joint loading environment is needed for the prevention of hip pain, and the reduction of the stress fractures and fall risks. Using the motion analysis and inverse dynamics methods, combined with musculoskeletal modelling, static optimization, and finite element (FE) femur model, the difference of femoral neck strains between stair ascent vs. descent, young vs. older populations was compared. A two-way repeated-measures MANOVA was applied to test the effect of age and stair direction on the femoral neck strains. The strains at the femoral neck cross-section were greater for stair descent than ascent for both age groups (mostly *P* = 0.001 to 0006) but there was no difference between age groups. In this study, femoral neck strains represented bone loading environment in more direct ways than joint reaction forces/moments or joint contact forces, the risk of hip pain, falls and stress fractures might be greater during stair descent than ascent. Possible preventative methods to reduce these risks should be developed in the future studies.

## Introduction

Bone fractures are among the injuries with most seriousness which could result in immobilization and cause other health issues which could be life-threatening. With the overall mortality rate of hip fractures at 14.0–21.6% and an estimated 6.26 million cases expected by 2050, fractures at the femoral neck play an important role in morbidity and mortality among people, especially the older populations^1,2^. Moreover, the estimated lifetime cost for all hip fractures in the United States and UK are staying high over years^3–6^. This musculoskeletal injury will play an increasing prominent role in the health and economical issues with an growing aging population^7–9^. Especially for stair ascent and descent, more hip join pain and falls are found during these activities for older population. Some research reported that about 30% of older population suffered at least one fall in life time, and 20–30% of falling led to the serious injuries including fractures, concussions etc^10–14^. Investigating the load environment for the femoral neck could be a useful way to investigate the risk factors of the stress fractures, femur pain and its related fall risks for some certain daily activities (e.g. stair navigation), which could be helpful to minimize further damage/pain at the hip joint or develop preventative measures to reduce the load on the femoral neck^15,16^.

A detailed analysis of the proximal femur load is necessary to understand the mechanisms of fractures and bone health. The hip joint forces and bending moments at the proximal femur during stair ascent and descent^17–21^ has been measured using instrumented hip prostheses, which is a direct measure that are invaluable and accurate. Stair descent produced greater hip joint contact forces and bending moments than ascent, but the invasive nature and limited sample size reduces the practicality of this protocol in most laboratory and clinical settings. Utilization of inverse dynamics and rigid body models allows the estimation of net joint moments and reaction forces during stair ascent and descent^22,23^, which found the greater hip moments for stair descent than ascent. But these methods neglected the effect of co-contracting muscles and fail to consider the size and material properties of the bone. Musculoskeletal modelling and muscle force estimation could be utilized with the 2-demensional simplified model to estimate femoral neck stresses for stair ascent and descent^24^, but the strain (deformation statues) estimation could not be performed with the 2-D model.

Finite element method (FEM) could be used to accurately model bone geometry, material properties, and femoral external loads given the appropriate load inputs for the femoral neck or other specific regions. This analysis could be used in a 3-dimensional (3-D) bone model so the strains (material deformation) for the femoral neck could be estimated. The 3-D Bone models could be derived from CT or MRI scans of bone specimens^25,26^, which provides more accurate information for the geometry and material properties to predict the load in a more realistic way. In the finite element models, loading moduli and compressive strength could be computed in each element of the model by using the correlation between calibrated CT scan and ash density, and another correlation between ash density and mechanical properties of trabecular and cortical bone^25,26^. These procedures could be performed by the commercially available computer programs (e.g. FEBio, Abaqus), which estimate stress and strain at each element of the bone model. Early applications for these models were load tests using artificial external load acting on the fixed points of the femoral head to simulate standing, walking or landing. The load conditions and failure criteria, bone failure theory were tested by these finite element models^27^. In vivo testing, finite element model with simplified muscle models could lead to significantly inaccurate estimation^28,29^, so the more complex and realistic load conditions (with full muscle models) should be needed to reflect the accuracy of the stress/strain estimation^30,31^.

Using finite element method to estimate the compressive (minimal principal) and tensile (maximal principal) strains on the femur model could show how much load is acting on different regions of the femur, especially on the femoral neck^32,33^. The strain estimation during daily activities could reveal the load environment of the femoral neck and analyze the load that could be responsible for injury or the proximal femoral pain syndromes. Moreover, gait changes as people age, and this aging effect combined with the changes in bone properties or muscular ability with age may lead to more risks of femoral neck fractures, hip pain or falls^14,15,34^.

Due to above aging effects, it is hypothesized that the femoral neck strain should be greater for older population than younger since older population suffered more hip pain and fall issues. The purposes of this study were to (1) compare peak compressive and tensile strains on the femoral neck between stair ascent and descent, (2) compare peak compressive and tensile strains between young and older groups, and (3) find the location of strain concentration (peak strain location) on the femoral neck.

## Results

The maximum femoral head deflections were lower than 1.7–2.0 mm for all the models, which were within a physiologically realistic range, in particular < 4 mm^14,29^. The main effect of stair direction was significant for both young and older groups (*P* < 0.001), while the main effect of age (*P* = 0.066) and the interaction effect of age by stair direction (*P* = 0.137) were not statically significant, a-posteriori power analysis was performed and showed the effect size f for MANOVA test was greater than 0.25.

**Figure 1:**
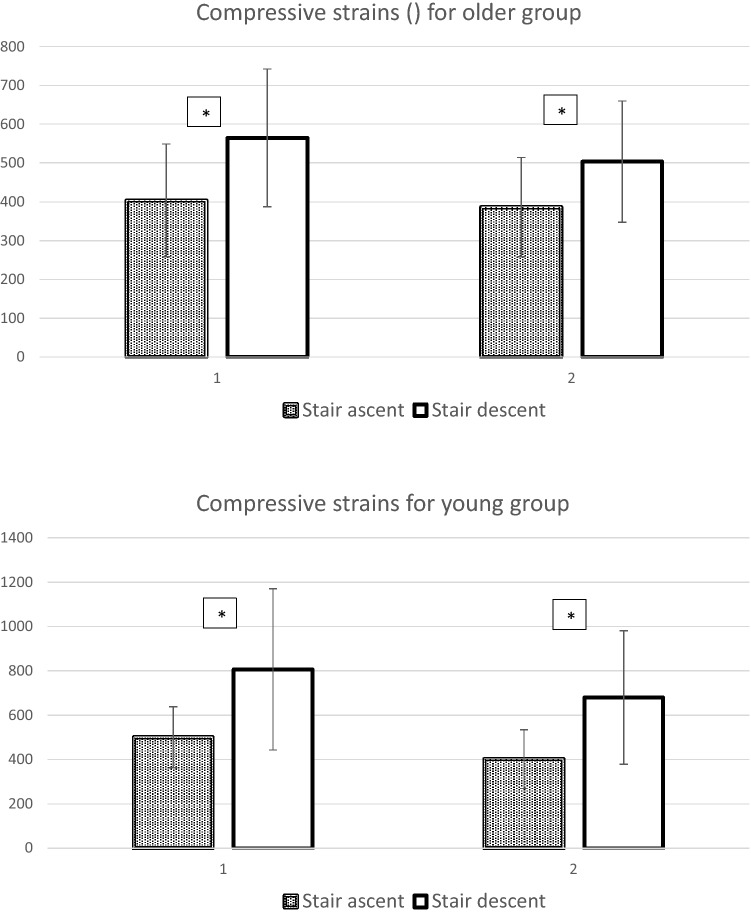
The averaged peak compressive strains (unit: µε) for older and young groups (Means ± SD). In Horizontal axis, 1 indicated peak 1, 2 indicated peak 2. *Stands for the significant difference comparing to stair descent (*P* < 0.01).

**Figure 2:**
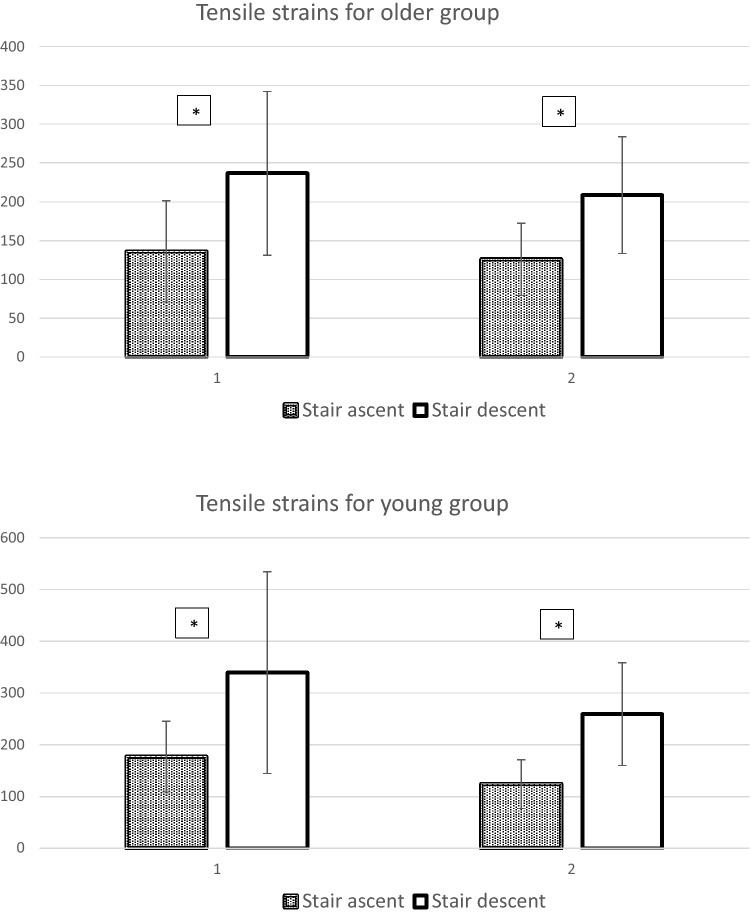
The averaged peak tensile strains (unit: µε) for older and young groups (Means ± SD). In Horizontal axis, 1 indicated peak 1, 2 indicated peak 2. *Stands for the significant difference comparing to stair descent (*P* < 0.01).

For older group, stair descent generated greater compressive strains than ascent (Peak 1: %DIFF = 28.5%, *P* = 0.001, Effect size = 0.98; Peak 2: %DIFF = 23.2%, *P* = 0.004, Effect size = 0.86), and descent had greater tensile strains than ascent (Peak 1: %DIFF = 42.5%, *P* = 0.006, Effect size = 0.80; Peak 2: %DIFF = 39.6%, *P* < 0.001, Effect size = 1.12), shown in Figs. 1 and 2. For young group, stair descent generated greater compressive strains than ascent (Peak 1: %DIFF = 38.0%, *P* < 0.001, Effect size = 0.89; Peak 2: %DIFF = 40.9%, *P* < 0.001, Effect size = 1.20), and descent had greater tensile strains than ascent (Peak 1: %DIFF = 47.9%, *P* = 0.003, Effect size = 0.75; Peak 2: %DIFF = 52.2%, *P* < 0.001, Effect size = 1.50), shown in Figs. 1 and 2.

Due to the non-significant main effect of age, there was no statistical difference between young and older groups for the femoral neck strains. The maximum compressive strains were -400 to -500 µε (stair ascent), -680 to -805 µε (stair descent) for young group; while for older group they were -385 to -405 µε (stair ascent), -500 to -565 µε (stair descent). For the tensile strains, strains for young group were 125 to 175 µε (stair ascent) and 260 to 340 µε (stair descent) while for older groups were around 125 to 135 µε (stair ascent) and 210 to 240 µε (stair descent).

**Figure 3:**
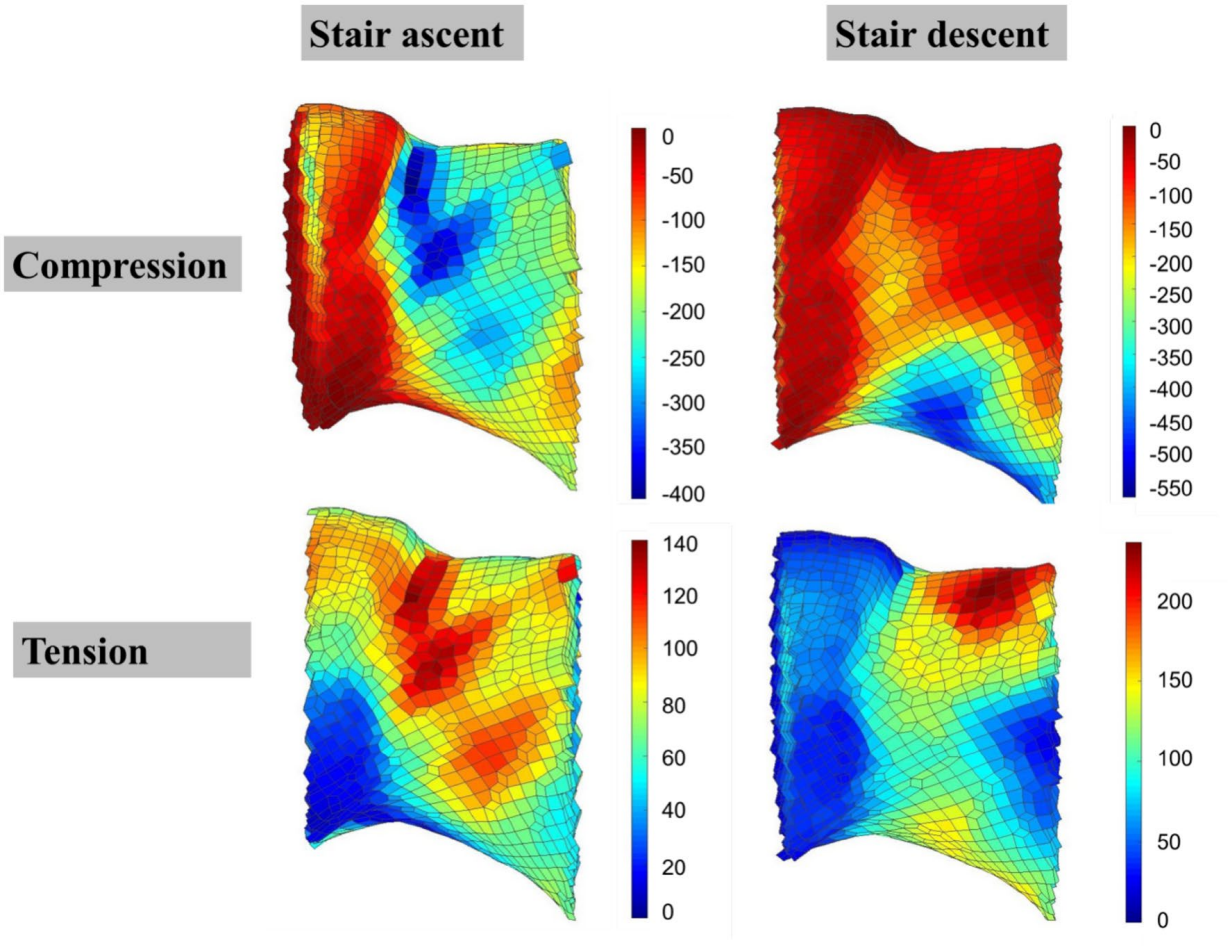
The strain distribution (unit: µε) for the femoral neck during the 1st peak, posterior view, older group (a representative participant). Positive values indicated tensile strains, negative values indicated compressive strains.

Figure 3 shows the location of strain concentration for both compressive and tensile strains. For stair ascent, the compressive strain concentrations were located at the mid-posterior surfaces at peak 1, and the upper-posterior surfaces at peak 2; the tensile strain concentrations were located at the upper to mid-posterior surfaces at peak 1, and the upper-posterior surfaces at peak 2. For stair descent, the compressive strain concentrations were at the inferior surface for descent at both peaks; the tensile strain concentrations were at the superior surface for descent at both peaks.

## Discussion

In this study, femoral neck strains were analyzed during stair ascent and descent using finite element method. Peak femoral neck strains were analyzed compared during stair navigation for young and older adults, which would be helpful to analyze the femoral neck load environment. This information could be used in understanding of the bone health, including prevention of femoral fractures, reduction of femoral pain and its related falls for the older population etc.

The compressive strains at the femoral neck were greater for stair descent than ascent, which indicated that the femoral neck suffered more compressive loads from stair descent than ascent. Greatest compressive strains for stair descent located at the inferior surface, which might mostly due to the large bending effect caused by the hip joint force acting on the femoral head^33,35,36^. In general, the hip joint force compressed the neck concave inferior during stair descent due to a relatively extended position of the hip and knee (maximal hip flexion angle from 8 to 12 degrees, 0 degree as the neutral position). Due to the same bending effect and a relatively flexed posture of the hip (maximal hip flexion angle from 45 to 50 degrees), the femoral neck concave was compressed more posteriorly for stair ascent.

Greater tensile strains at the femoral neck were found for stair descent, which mainly located at the superior surface. This could be caused by the tensile bending effect at the superior surface of the neck due the hip joint force with a relatively extended position of the hip. For stair ascent, the greater tensile strains would have been located around the anterior surface of the femoral neck due to this bending effect and the flexed posture of the hip. However, the tensile strain concentrations were at the upper to mid-posterior surfaces, which were closed to the locations of compressive strain concentrations. The greater adductor muscle activities during stair ascent could be one explanation: this effect could cancel out the tensile effect caused by the hip joint force^24,37^, which could be a protective method for the superior surface of femoral neck. The Poisson ratio was setup as 0.3 for each element in the model. For each element, greater compressive strain at in one direction would cause greater tensile strains in the lateral directions. The ratio of tensile and compressive strains at the posterior area was approximately 0.3 (tensile/compressive), so these tensile strain concentrations could be partially caused by the large compressive strains.

Comparing to stair ascent, less muscular activities for the hip/knee extensor and hip abductor muscle were found for stair descent in this and prevous stuides^24,37^. the decrease of muscle force during stair descent might minimize the muscular protection to the femur^24,37^. The principal strains at the femoral neck during stair ascent and descent were much lower than the walking tests from Anderson^32^ but similar with the tests from Edwards^38^, this difference might partially due to 25% lower gait speed (0.84–0.92 m/s) during stair ascent/descent than walking, or due to difference in the assigned material property/stiffness.

The result did not support the hypothesis that older population had more femoral neck strains than young population. One explanation for this phenomenon could be a much slower pace for older participants during stair navigation: much longer ground contact time and slower walking speed was found for older participants in this study, which could result in lower ground impact during stair ascent and descent. Moreover, the age requirement for the older groups was not same as older population (usually defined by more than 65 yrs): most participants were from 57 to 62 yrs, the oldest one was 65 yrs. For young group, some participants were landed carefully and some younger ones (18–20 yrs) were landed heavily, this could cause the higher variability in strains during stair descent for young group (Table 1).

**Table 1:** Means ± SD of peak strains (µε) for the femoral neck for young and old groups during stair ascent and descent, strains were averaged among participants within the group. * stands for the significant difference comparing to stair descent (*P* < 0.01).

	Stair ascent		Stair descent	
Strain type	Peak 1	Peak 2	Peak 1	Peak 2
	**Older group**			
Compressive	− 403.6 ± 144.8*	− 386.3 ± 127.7*	− 564.7 ± 177.2	− 503.3 ± 155.9
Tensile	136.1 ± 65.3*	126.0 ± 46.5*	236.7 ± 105.3	208.7 ± 75.1
	**Young group**			
Compressive	− 500.1 ± 137.5*	− 401.8 ± 132.7*	− 806.6 ± 363.6	− 679.3 ± 301.2
Tensile	176.8 ± 68.5*	123.9 ± 46.7*	339.3 ± 195.0	259.0 ± 99.3

Both compressive and tensile strains analyzed in this study could be presented as direct variables related to the loads that cause hip pain, falls and bone damage or fracture. The results showed that stair descent produced more femoral neck strains than ascent, which indicated that the risk of hip pain, falls and stress fractures could be higher for stair descent, more alternative/preventative methods to reduce stress and strain effectively should be developed for older population.

The method used in this study still remained some limitations: (1) the finite element models (a 99 year old cadaver) do not incorporate any subject-specific imaging to provide information on bone geometry or the distribution of bone density, which might lead to lower loading moduli than ultrasound or unloading measurements^39^; (2) using individualized CT or MRI scans could give more accurate prediction; (3) isotropic material properties could lead to overestimation of the tissue stiffness^40^; (4) potential errors for the estimation from the musculoskeletal model and finite element models may still remain. For the future studies, a more individualized femoral neck model (based on CT scans and anisotropic material properties) and subject-specific musculoskeletal model (based on ultrasound) could be used in the testing. With more accurate information for each individual, the individualized preventative methods or training plan could be developed to enhance the health status of older population.

## Conclusion

For both young and older groups, stair descent produced more femoral neck strains than ascent, which indicated that the risk of hip pain, falls and stress fractures could be higher for stair descent for older population. More alternative/preventative methods (slower speed, change gait type etc.) to reduce stress and strain on the femoral neck should be developed for older population to avoid hip pain, falls and stress fractures during stair navigation.

## Methods

In this study, all methods were carried out in accordance with relevant guidelines and regulations from the Biomechanics Laboratory, Department of Kinesiology, Iowa State University. All experimental protocols were approved by the Iowa State University Human Subjects Review Board.

To perform a two-way repeated-measures MANOVA, it was determined that a minimum of 34 participants in total would be needed to detect differences in strains between stair ascent and descent, between young and older groups with a medium effect size of 0.25 for with a power of 0.80 (GPower 3.1). For this study, seventeen older adults and twenty young adults who were free from lower limb injuries volunteered to participate (Table 2). Before participation, they signed a written informed consent document that had been approved by the Iowa State University Human Subjects Review Board.

**Table 2:** Subject information: gender, age, body weight and height; XX (X) stands for Mean (SD).

Group	Old		Young	
Gender	Male	Female	Male	Female
Number	7	10	10	10
Age (yrs)	60	57	23	23
	(6)	(5)	(3)	(3)
Weight (kg)	75	67	80	62
	(14)	(8)	(14)	(10)
Height (m)	1.73	1.67	1.76	1.70
	(0.05)	(0.05)	(0.07)	(0.07)

Body mass, height, and anthropometric measures for the right lower extremity segments (segmental lengths, widths, and circumferences) were measured. Eighteen reflective markers were placed on anatomical landmarks of the trunk, pelvis, and right lower extremity with a minimum of 3 markers per segment. Toe, heel and lateral foot markers were placed for the foot segment; anterior leg, posterior leg and ankle markers were placed for the leg segment; anterior thigh, right hip and knee markers were placed for the thigh segment; both right and left hip, both right and left Anterior superior iliac spine (ASIS) and sacrum markers were placed for the pelvis segment; medial and lateral ankle markers could be used both in the foot and leg segments, medial and lateral knee markers could be used both in the leg and thigh segments. All anthropometric measurements and marker placements were performed by the same researcher. A static trial was collected with the subject in anatomical position to estimate joint center locations by the markers on the joints and then medial side markers of the lower extremity were removed. All subjects performed five trials of stair ascent and five trials of descent (three-step staircase, height of each stair: 19 cm) at their normal comfortable speed without any external support (handrail etc.). For each trial, the participant used the left foot to take the 1^st^ step, then took the 2^nd^ step with the right foot, which landed on the force platform. AMTI force platforms (1600 Hz, AMTI, Watertown, MA) were placed on the two lower stairs to measure ground reaction forces. Motion data were collected using an 8-camera system (160 Hz, Vicon MX, Centennial, CO).

Ground reaction forces and motion data were filtered using a low-pass Butterworth filter with a cutoff frequency of 6 Hz^41^. The stance phase cycle for stair ascent/descent began with the first contact of the right foot on the force platform and finished with its toe-off. All gait cycles were normalized into a percentage of the stance phase. A rigid body model was used with inverse dynamics procedures to estimate three-dimensional joint moments and reaction forces at the ankle, knee, and hip. Segment masses, center of mass locations, and moments of inertia were obtained^42^. Joint moments and reaction forces were calculated in the global coordinate system and then transformed into the coordinate system of the proximal segment at each joint.

An individually scaled musculoskeletal model based on the joint and muscle definitions of Arnold^43^ was implemented in Matlab to estimate the dynamic muscle–tendon length, velocity adjusted maximal muscle forces, muscle moment arms and orientations for 44 lower limb muscles. The three dimensional segment angles obtained during the trials were used in the model. Static optimization was used to select a set of muscle forces that minimized the sum of the squared muscle stresses^44^ and balanced using the sagittal plane hip, knee and ankle moments, frontal plane hip moment and the transverse plane hip and ankle moments. Solutions were also constrained by the maximal dynamic muscle forces estimated with the musculoskeletal model.

$$Min {\sum_{\mathrm{i}=1}^{44}(\mathrm{F}}_{\mathrm{i}}/{\mathrm{A}}_{\mathrm{i}}{)}^{2}$$ Subject to: $${\mathrm{r}}_{\mathrm{ij}}\times {\mathrm{F}}_{\mathrm{i}}={\mathrm{M}}_{\mathrm{j}}$$ $$0\le {\mathrm{F}}_{\mathrm{i}}\le \mathrm{Max\,\,dynamic }\,\,{\mathrm{F}}_{\mathrm{i}}$$

For the ith muscle: F_i_ is estimated muscle force, A_i_ is the cross-sectional area, r_ij_ is the moment arm for the jth joint moment, and M_j_ is the jth joint moment.

The hip joint contact forces were calculated by the sum of the hip joint reaction forces and the muscle forces across the hip joint. The 3-demensional hip joint contact forces were acting on the femoral head of the femur model.

The finite element model for the whole femur was provided by VAKHUM database^45^. The model was developed by the clinical CT scans for the femur from a female cadaver (age: 99-yr; mass: 55 kg; height: 1.55 m), and the apparent density was calculated according to Schileo^30^. The acquisition setting of the CT scan was 120 kVp and 200 mAs, and images were reconstructed with a slice thickness of 2.7 mm and an in-plane pixel resolution of 0.840 mm. The finite element model contains 104,945 linear hexahedral elements with 115,835 degrees of freedom (or nodes). The default element edge length is 2.0 mm. Principal stresses, and principal strains will have less than 3% change when increasing element edge length from 2.0 to 3.0 mm, which guaranteed the adequate convergence at the H-refinement.

The geometry of femur model was scaled by the individual thigh length in longitudinal direction, and then scaled by the length·diameter^2^ ∝ body mass in radial direction according to McMahon^46^. The gender specific correlations between Young’s modulus and age was developed from Burstein^47^ and the calculated Young’s modulus data from the original cadaveric femur. The isotropic material property (Young's modulus and Poisson's ratio) of whole femur model was justified uniformly by these correlations for each participant. Each element was assigned to one of 286 linear-elastic material properties. The density-elasticity relationship was based on mechanical testing data of femoral neck^48^:

E = 6850ρ_app_^1.^^49^, where E is the loading moduli in MPa, and ρ_app_ is the apparent density in g/cm^3^; all materials were assigned a Poisson’s ratio of 0.3.

**Figure 4:**
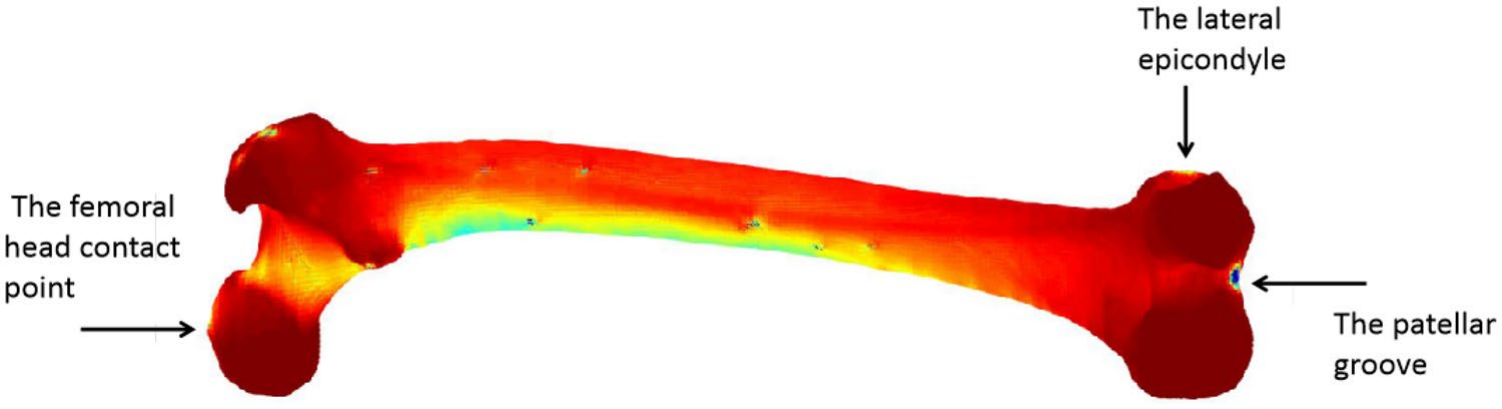
3-dimensional model for femur and the boundary condition locations.

The finite element and musculoskeletal models were aligned into a common local coordinate system. 27 femoral muscle insertion locations from Arnold’s model were mapped to surface nodes of the finite element model. The finite element model was physiologically constrained at the lateral epicondyle in the anterior–posterior direction; center of the patellar groove in the anterior–posterior, medial-laterial and vertical directions; and the femoral head contact point in the anterior–posterior, medial-laterial directions^29^, shown in Fig. 4. All muscle forces and joint contact forces were applied as point load for the femur model.

The compressive and tensile strains at the whole femur were analyzed during both hip joint contact force peaks. The whole femoral neck cross-sections were extracted for each subject and all forces were applied as point loads and resulting strain concentrations were removed from further analysis by discarding nodes and elements in the immediate vicinity of load application^48^.

The independent variables were the direction of travel (ascent vs descent) and the age group (young vs older), the dependent variable were compressive (minimal principal) and tensile (maximal principal) strains. The strain was estimated at the two time points during the stance phase that corresponded with the peak hip joint contact force values from the bimodal curves in the contact force by time plot. The averages of 5 trials for each direction were used for statistical analysis. A two-way repeated-measures MANOVA was used to compare the differences between the directions (ascent vs descent) and between the age groups (young vs older) as well to test for an age by direction interaction (SPSS, IBM Corp). Univariate ANOVAs were performed given a significant multivariate statistic. If sphericity was violated a Greenhouse–Geisser correction was performed. Pairwise t-tests were used to compare the maximum strains on the femoral neck between stair ascent and descent during the 1st and 2nd hip contact force peaks, for young and older group separately. The alpha level was set at 0.05 for all statistical tests.

The compressive and tensile strains are presented in the femoral neck cross-section (Table 2). The percentage of strain differences (%DIFF) were calculated using the follow equation (Strain_SD_ = strain for stair descent, Strain_SA_ = strains for ascent):

$$\% DIFF = \frac{{Strain_{{SD^{{}} }} - Strain_{SA} }}{{Strain_{SD} }}$$

## Supplementary Information

Supplementary Information 1.

Supplementary Information 2.

## Data Availability

The original/raw datasets generated and analysed for the current study are available from the corresponding author on reasonable request. The datasets under the statistical analysis was uploaded as the supplementary files.
